# Fusion of CNN and swin transformer for robust lung sound classification

**DOI:** 10.1007/s13534-026-00616-6

**Published:** 2026-09-23

**Authors:** Asefa Natnale Sitotaw, Myungrae Hong, Sunghoon Im, Daeshik Kang

**Affiliations:** 1 Mechanical Engineering Department, Pohang University of Science and Technology (POSTECH), 77 Cheongam-ro, Pohang-si, Gyeongsangbuk-do 37673 Republic of Korea; 2 Agency for Defense Development, Daejeon, 34186 Republic of Korea

**Keywords:** Fusion architecture, Balanced detection, Pathological events, CNN, Swin transformer

## Abstract

Automatic lung sound classification plays a pivotal role in the diagnosis of chronic respiratory diseases, enabling early screening and informed clinical decisions. However, evaluating model performance on imbalanced datasets, such as the ICBHI 2017, remains challenging. As a result, existing studies have focused on improving overall accuracy or average score, while paying limited attention to achieving balanced detection between pathological and healthy events. From a clinical perspective, such an imbalance introduces systematic diagnostic bias, manifested as over-detection or under-detection of disease or abnormal lung sounds, even when high-performance metrics are reported. This study evaluates 1D-CNN, 2D-CNN, Swin Transformer, and a fusion CNN–Swin Transformer model, including its pretrained variant, under the official evaluation protocol. The fusion model combines CNN-based local time-frequency extraction and global contextual modeling using the Swin transformer. The results show that the fusion architecture and its pretrained variant across all ICBHI tasks have a balanced detection between true positive and true negative cases. Improvements in sensitivity up to 5.8% and harmonic score up to 3.5% are observed in certain tasks, reflecting strong generalization in detecting pathological events while maintaining specificity.

**Supplementary Information:**

The online version contains supplementary material available at https://doi.org/10.1007/s13534-026-00616-6.

## Introduction

Chronic respiratory diseases (CRD) such as asthma and COPD are preventable diseases. However, they remain the five leading causes of death and illness in the European region, accounting for nearly 400,000 deaths annually, with COPD alone responsible for 80% of these deaths [1]. Globally, respiratory diseases such as asthma, COPD, lower respiratory tract infection (LRTI), tuberculosis, and lung cancer rank as the third leading cause of death. Effective strategies for disease prevention, early detection, monitoring, and treatment are mandatory to address these problems [2–4]. Pulmonary auscultation with a stethoscope remains the most widely used clinical method for assessing patient respiratory sounds. Due to its simplicity, efficiency, and low cost, it has long served as a valuable tool for evaluation and ongoing monitoring in clinical environments [4]. However, since classical auscultation depends on clinician diagnostic ability (hearing) and subjective judgment (diagnostic interpretation), its performance cannot be objectively improved or optimized [5]. To address this limitation, research on automatic detection of respiratory sounds has been conducted for many years, with several notable precedents. Recent advances have increasingly focused on the use of artificial intelligence (AI) and deep learning to improve diagnostic accuracy and effectiveness [3].

Despite progress, automatic lung sound auscultation remains challenging, particularly in the ICBHI 2017 datasets, which are characterized by high class imbalance, variable recording, and inconsistent sampling rates [6]. Earlier studies applied shallow machine learning (ML) models such as convolutional neural network (CNN)-based architectures to these datasets [7, 8]. CNNs are effective in extracting local features [5, 8, 9]. However, their architecture has limitations in capturing broader spatial relationships. In recent years, attention-based architectures (Transformers) have emerged as powerful alternatives to traditional convolutional models [10]. These models can dynamically weigh the importance of different feature regions, allowing them to capture long-range dependencies and complex global relationships within input space. This capability motivated researchers to adopt a Transformer-based architecture to handle the challenging ICBHI problem [8, 10]. Inspired by these motivations, Hussain et al. [11] proposed a hybrid of CNN and Transformer to improve Parkinson’s disease (PD) classification for brain MRI images. The authors argue that previous MRI-based PD methods rely on CNN-based representations. However, the lack of clear MRI biomarkers and the extremely subtle structural differences between PD and healthy controls limit the CNNs’ ability to capture long-range dependencies. To overcome this, they used a hybrid CNN and Swin Transformer feature fusion at each stage of feature extraction and showed that their model achieves superior accuracy and robustness compared with existing methods. Moreover, unlike respiratory sound analysis, their task does not involve time-dependent acoustic patterns for which sequential and contextual information across respiratory events is critical in evaluating the true benefit of Swin Transformer-based representation learning.

Previous studies have significantly advanced respiratory sound classification and demonstrated strong overall performance. However, clinical reliability requires more than high accuracy alone, particularly when sensitivity and specificity are not equally balanced. In practical diagnosis, models should reliably recognize both normal and pathological respiratory sounds. Therefore, stability, reproducibility, and balanced diagnostic performance using heterogeneous patient data remain essential for real-world clinical adoption [12]. Balanced sensitivity (Se) and specificity (Sp) are critical in biomedical classification. When sensitivity is prioritized without sufficient specificity, normal cases can be incorrectly classified as abnormal, leading to excessive false-positive alarms. In contrast, when specificity is prioritized without sufficient sensitivity, abnormal cases can be incorrectly classified as normal, resulting in missed clinically relevant pathological events. Therefore, balanced detection is necessary to reduce false or non-actionable alarms while preserving clinically actionable detections [13, 14].

Given this limitation as the main motivation, the objective of this study is to address the diagnostic imbalance observed in respiratory sound classification, particularly the unstable trade-off between sensitivity (Se) and specificity (Sp). Rather than focusing only on improving a single overall performance score, this work emphasizes the need to stabilize diagnostic balance using clinically meaningful evaluation metrics. The CNN–Swin Transformer fusion framework was selected for this purpose because the se–sp imbalance in respiratory sound classification is mainly caused by two competing errors: missing subtle abnormal acoustic events, which reduces sensitivity, and misclassifying noisy or non-pathological respiratory patterns as abnormal, which reduces specificity. The CNN branch captures local spectro-temporal patterns such as crackle- and wheeze-related textures. The Swin Transformer branch provides hierarchical window-based attention to model broader contextual dependencies across the spectrogram. CNNs with attention-based models are fused to combine fine-grained local representations with long-range contextual information for improved diagnostic performance [10, 11, 15]. Therefore, in this study, the CNN–Swin Transformer fusion architecture is chosen to improve the diagnosis of the noisy and unbalanced ICBHI dataset. This approach is evaluated against representative baseline models, including a 1D-CNN designed to capture temporal features, a 2D-CNN for spectro-spatial features, and a Swin Transformer for global contextual dependency. In addition, a pretrained fusion variant is investigated to assess whether prior feature representations can improve diagnostic performance. The experimental assessment is evaluated in multiple classification tasks, including both anomaly-level and disease-level classification, under a strict 60/40 patient-wise split. This multi-task validation uses a broader assessment of model robustness and the ability to handle class imbalance in respiratory sound classification.

## Related work

Related studies that address the challenges of the ICBHI datasets are discussed in detail in this section. The review is organized into two main categories: CNN-based approaches and self-attention-based approaches.

In the CNN-based literature, Demir et al. [16] introduced a CNN architecture that integrates max- and average-pooling branches to improve feature representation and overall classification performance. However, their evaluation criteria did not follow the official ICBHI protocol, which limits the comparability of their results with other literature. In contrast, Pham et al. [4] conducted a systematic study of the effects of preprocessing and augmentation strategies to improve performance while strictly following the official ICBHI evaluation protocol. They also proposed a CNN-based model with a mixture-of-experts head, enhanced using mixup and teacher-student distillation. Their result shows that, in the official 60/40 patient-wise data split, significant gains were achieved in disease-level classification–84% in both the three-category and two-category tasks. However, the four-class respiratory cycle recognition task remained challenging, achieving an AS of only 47% and revealing a large imbalance between Se (26%) and Sp (68%). Gairola et al. [17] extend this line of CNN-based approaches by using a pretrained ResNet-34 backbone and applying data enhancement strategies to improve generalization and overall performance. Although they evaluated their method on the four-class and two-class tasks, they used a random data split rather than the patient-wise split required by the official ICBHI protocol. In this random split for the four-class task, the model achieves an average score (AS) of 56.2%. However, the result shows a clear imbalance between Se (40.1%) and Sp (72.1%). Wu et al. [18] move beyond single-branch CNNs by proposing a dual-stream Bi-ResNet that integrates STFT and wavelet features, resulting in a substantially improved four-class accuracy of 77.81%. Wang et al. [19] also used a ResNet-34 backbone for the four-class classification task. Unlike the studies by [16, 17], they strictly followed the official protocol. Using extended 7-second cycles, their model achieved an AS of 50.2%. However, a major imbalance remained, with a high Sp of 75.3% and a very low Se of 25%. Other studies have sought to mitigate the imbalance of the ICBHI datasets by improving optimization stability and use of pretrained models. Wanasinghe et al. [20] train several CNN models by stacking three complementary acoustic features–Mel-spectrogram, MFCC, and Chromogram–by combining the Mendeley and ICBHI datasets. In the ten-class disease classification task, their best-performing model achieved 91% accuracy. Polanco-Martagón et al. [12] trained pretrained CNN backbones (VGG16/19, InceptionV3, MobileNetV2, and ResNet152V2) using the official 60/40 patient split and found that the fine-tuned ResNet-152V2 achieved the highest performance with an average score of 54.1%. However, as their result shows, the pretrained CNN tends to exhibit bias towards high specificity at the expense of sensitivity, potentially leading to missed pathological events. To address this imbalance, they proposed a dual-stream VGGish variant that achieved balanced detection of healthy and pathological events.

**Fig. 1:**
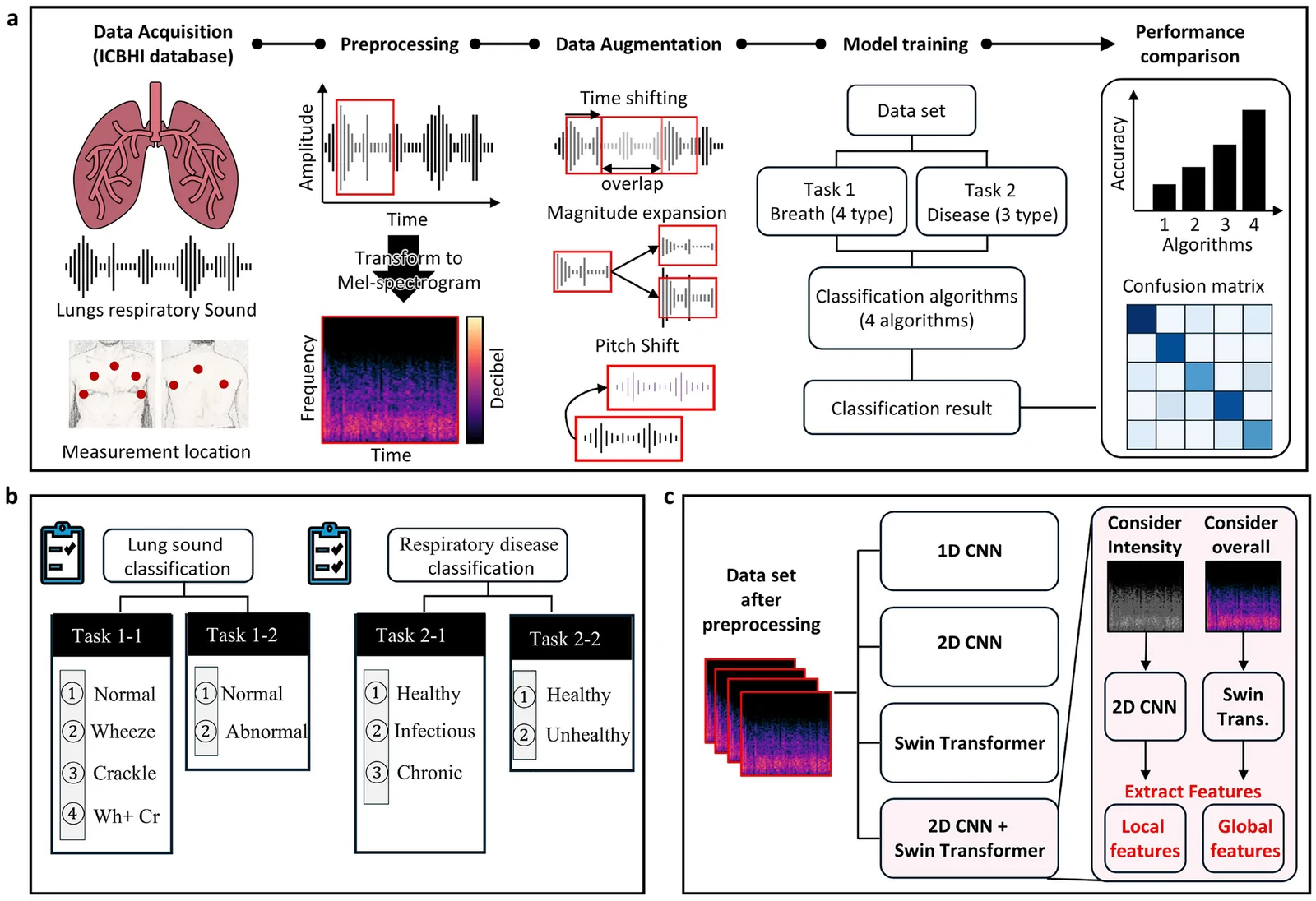
Overall workflow and task design **a** End-to-end pipeline: collection, preparation, modeling, assessment; **b** Model architecture: CNN, Swin, fusion; **c** Task taxonomy: Task 1(anomaly) and Task 2 (disease)

In the self-attention approach, several works utilize self-attention architectures to capture complex lung sound patterns. The Vision Transformer (ViT), a prominent attention-based architecture derivative, effectively captures global contextual relationships across the input space. However, its performance depends on the size of the datasets and computational resources. Neto et al. [9] applied a ViT model for a 4-class task and achieved high AS and HS in the standard metrics. However, when evaluated using the ICBHI metric, the performance dropped to an AS of 43.13% and an HS of 41.4%, reflecting ViT’s limitation for limited data. Nair et al. [10] continued this line of attention-based approaches by integrating EfficientNet-B0 with a lightweight attention head and broad pre-processing. Although training is time-consuming, inference is significantly faster than in other pretrained models. In their random 80/20 train–test split, they reported validation accuracy of 99.8%. Bae et al. [21] also applied attention-based modeling following the ICBHI protocol. They used a pretrained Audio Spectrogram Transformer (AST) and introduced a patch-mixing strategy based on random patch swapping instead of mixing the entire spectrogram. They reported a 4.1% increase in AS, indicating improved model robustness. However, a major imbalance remained, with a high Sp of 86.66% against a very low Se of 43.07%. Building on previous Transformer-based studies, Wu et al. [8] propose a fully self-attention-based approach and compare FNN, CNN, AST, and a dual-input AST (fusing spectrogram and log-Mel features) under the same ICBHI train/test split and metrics. They also studied respiratory-cycle-based segmentation as an alternative to fixed-window-based segmentation. Their results showed that the dual-input AST with cycle-based segmentation outperformed all baseline models, achieving an AS of 52.5% and an HS of 50.8%. Overall, the reviewed Transformer-based models achieve strong performance when sufficient data is available. However, the need for large-scale data and high computational cost deters their effectiveness [22]. As a result, large-scale augmentation or initialization with pretrained data is necessary. In general, the reviewed CNN and attention-based models tend to achieve high specificity at the expense of sensitivity, potentially leading to missed pathological events. This is especially prominent for models that follow the strict ICBHI evaluation protocol. Although transformer-based models offer strong detection performance, their effectiveness depends on sufficient data. To overcome this limitation, we employ the Swin Transformer, which uses hierarchical modeling in which the input is divided into non-overlapping windows and processed using shifted window-based multi-head self-attention [22]. To address the imbalance problem, we use a fusion architecture that integrates CNN with SWIN transformers, combining spectro-spatial patterns and global contextual relationships. Furthermore, to strengthen feature representation and address data inefficiency, we integrate pretrained components into our hybrid design, aiming to achieve a more balanced sensitivity-specificity profile.

## Methods

Figure 1 illustrates the overall pipeline of the proposed lung sound classification framework. The process begins with data acquisition, followed by preprocessing and data augmentation. The recordings are then converted to Mel-spectrogram representation for model training and performance comparison. As shown in Fig. 1b, Task 1 focuses on abnormal lung sound detection, whereas Task 2 targets respiratory disease classification. For all tasks, different training architectures–including 1D-CNN, 2D-CNN, Swin Transformer, and their fusion (Fig. 1c)–are analyzed.

**Table 1:** Data distribution for ICBHI task respiratory cycles (Task 1) and disease classes (Task 2)

Task 1		Task 2	
Respiratory Cycles	No. of Samples	Disease Class	No. of Patients
Normal	3642	Healthy	26
Wheeze	886	Chronic disease	72
Crackle	1864	Infectious disease	28
Both	506		

### ICBHI dataset

We base our experiment on the publicly available ICBHI challenge dataset [23]. The raw data comprises 920 annotated recordings collected from 128 patients, totaling approximately 5.5 h of audio data. Each recording was acquired from different chest locations, as illustrated in Fig. 1a. Within these recordings, a total of 6898 respiratory cycles are labeled by clinical expertise and categorized as normal, wheeze, crackle, and both (crackle + wheeze). Furthermore, each record is annotated with the patient’s respiratory diagnosis, such as Healthy, COPD, Asthma, Bronchiectasis, Upper respiratory tract infection (URTI), Lower respiratory tract infection (LRTI), Pneumonia, and Bronchiolitis.

### Proposed tasks

Using the cycle-level annotations as shown in Fig. 1b and summarized in Table 1, we tackle Task 1-1 with four classes (normal, wheeze, crackle, and both). For Tasks 1-2, we reclassify the breath conditions into two categories by regrouping wheeze, crackle, and both into the abnormal (or anomalous) category, forming a binary classification against the normal class. Furthermore, using recording-level annotations as depicted in Fig. 1b and summarized in Table 1, we reclassify the disease conditions as healthy, chronic diseases (including COPD, asthma, and bronchiectasis), and infectious diseases (including URTI, LRTI, pneumonia, and bronchiolitis). With this regrouping, we address Task 2-1. For Task 2-2, we further combine chronic and infectious diseases into a single unhealthy class, resulting in a binary classification task: healthy versus unhealthy. For all experiments, we strictly follow the ICBHI standard protocol, which divides the data into non-overlapping 60% training and 40% test sets based on the patient-level separation [23]. Specifically, for disease classification, data splitting is performed at the patient level, where audio recordings are divided into training and test sets based on patient identity. Lung sound classification is performed at the respiratory-cycle level. However, respiratory-cycle segmentation is applied only after the ICBHI patient-wise train/test split. This ensures that all respiratory cycles or segments derived from the same patient’s recordings are assigned to the same split, thereby preventing data leakage during evaluation.

### Evaluation metrics

The evaluation of both the baseline models and the proposed approach strictly followed the official ICBHI evaluation criteria. The sensitivity (Se), specificity (Sp), average score (AS), and harmonic score (HS) are defined as follows [23]. For Task 1-1 (four classes),

1 $$\begin{aligned} Se = \frac{\hat{N}_{crackle} + \hat{N}_{wheeze} + \hat{N}_{both}}{N_{crackle} + N_{wheeze} + N_{both}} \end{aligned}$$

where $\hat{N}$ and *N* refer to the correctly predicted count and the true counts of abnormal cycles, respectively. Tasks 1–2 are evaluated by considering the binary version of Task 1–1 as described in [4]. *Sp* in both cases can be defined as follows:

2 $$\begin{aligned} Sp = \frac{\hat{N}_{normal}}{N_{normal}} \end{aligned}$$

For Task 2, recordings are classified into healthy, chronic, and infectious classes. The sensitivity is defined as follows:

3 $$\begin{aligned} Se = \frac{\hat{N}_{chronic} + \hat{N}_{infectious}}{N_{chronic} + N_{infectious}} \end{aligned}$$

Similarly, Task 2-2 is analyzed considering the binary version of Task 2-1, following [2]. We then define:

4 $$\begin{aligned} Se = \frac{\hat{N}_{Healty}}{N_{Healty}} \end{aligned}$$

For each task, after evaluating the sensitivity and specificity, the AS and HS of the test results are calculated as follows [22]:

5 $$\begin{aligned} AS = \frac{{Se+Sp}}{{2}}, HS = \frac{{2SeSp}}{{Se+Sp}} \end{aligned}$$

In addition to the standard evaluation metrics recommended by the ICBHI challenge, we further employed widely used biomedical classification metrics, including the Area Under the Receiver Operating Characteristic Curve (AUC) and Macro F1-score, to provide a more comprehensive assessment of the proposed model’s discriminative capability and class-balanced performance. For multi-class classification, the micro-average ROC curve was additionally computed by aggregating the contributions of all classes to evaluate the overall classification performance across the entire dataset.

6 $$\begin{aligned} AUC=\int _{0}^{1} TPR(FPR)\,d(FPR) \end{aligned}$$

Where the micro true positive rate (TPR) and false positive rate (FPR) are calculated as follows [24]:

7 $$\begin{aligned} TPR_{\text {micro}}= \frac{\sum _{i=1}^{C}TP_i}{\sum _{i=1}^{C}(TP_i+FN_i)} \end{aligned}$$

8 $$\begin{aligned} FPR_{\text {micro}}= \frac{\sum _{i=1}^{C}FP_i}{\sum _{i=1}^{C}(FP_i+TN_i)} \end{aligned}$$

The macro F1-score, which provides a balanced evaluation of classification performance by assigning equal importance to all classes regardless of class distribution, is calculated as [24]:

9 $$\begin{aligned} Macro\text {-}F1=\frac{1}{C}\sum _{i=1}^{C}F1_i \end{aligned}$$

where $F1_i$ represents the class-wise F1 score of the $i^{th}$ class, calculated from the corresponding precision and recall values as follows:

10 $$\begin{aligned} F1_i=\frac{2 \times Precision_i \times Recall_i}{Precision_i+Recall_i} \end{aligned}$$

Established performance metrics, such as MCC, macro-F1, and AUC, primarily quantify overall classification performance. However, they do not explicitly capture the asymmetry between sensitivity and specificity, particularly under the ICBHI evaluation protocol, where sensitivity and specificity are defined as separate task-specific measures using formulations that differ from conventional binary classification. Such results can be misleading, as the model may appear to achieve high aggregate performance while exhibiting a strong imbalance between disease detection and healthy recognition, a phenomenon observed in many studies that use the ICBHI dataset as a benchmark. From a clinical perspective, such an imbalance introduces systematic diagnostic bias, manifested as over-detection or under-detection of disease or abnormal lung sounds. For this reason, we adopted a new metric, the Diagnostic Balance Index (DBI), which rewards models when sensitivity and specificity are closer, thereby reducing diagnostic bias. A DBI value close to 1 indicates high diagnostic balance, whereas values near 0 indicate strong bias. The DBI is defined as:

11 $$\begin{aligned} DBI =1- \frac{{|Se-Sp|}}{{Se+Sp}} \end{aligned}$$

### Preprocessing

Figure 1a shows the end-to-end process pipeline. Because the raw recordings were acquired at varying sampling rates ranging from 4 to 44.1 kHz, all signals were resampled to 10 kHz and converted to mono. During poly-phase resampling, edge padding is applied. Moreover, cycle durations are standardized to fixed 5-second windows. To further improve signal quality, a 10th-order Butterworth-bandpass filter (50–2000 Hz) with zero-phase processing is applied for denoising. To mitigate data imbalance and improve the model’s generalization, pitch shifting (-4 to 4 semitones), loudness (-6 dB to 6 dB), and mild Gaussian noise were applied as data augmentations. Finally, to lower sensor-related variability and stabilize model training, the amplitude is normalized using per-segment z-score scaling.

### Feature extraction

After setting all respiratory cycles to 5-second segments, we compute a power spectrogram via the Short-Time Fourier Transform (STFT) with a 1024-point FFT, a Hann window, and a 28-sample hop. The resulting spectrum is then mapped to a Mel filter bank with 128 bands, followed by logarithmic scale conversion. Because the cyclic event segmentation is done by clinical experts and a log-mel spectrogram with short hop sizes is employed, the sensitivity to absolute temporal alignment is minimized. These strategies improve the robustness of respiratory cycle analysis and reduce distortions introduced by zero padding or truncation, particularly for short-duration crackle sounds. The resulting Mel spectrogram is resized to 224*× *224 pixels, a resolution taken to ensure compatibility with Swin Transformer and pretrained backbones such as EfficientNet. The resulting spectrogram is then standardized per segment using z-score normalization, clipped to *± 3* standard deviations, and stacked into three channels with shape (3, 224, 224) to match the RGB-like input expected by the models.

**Fig. 2:**
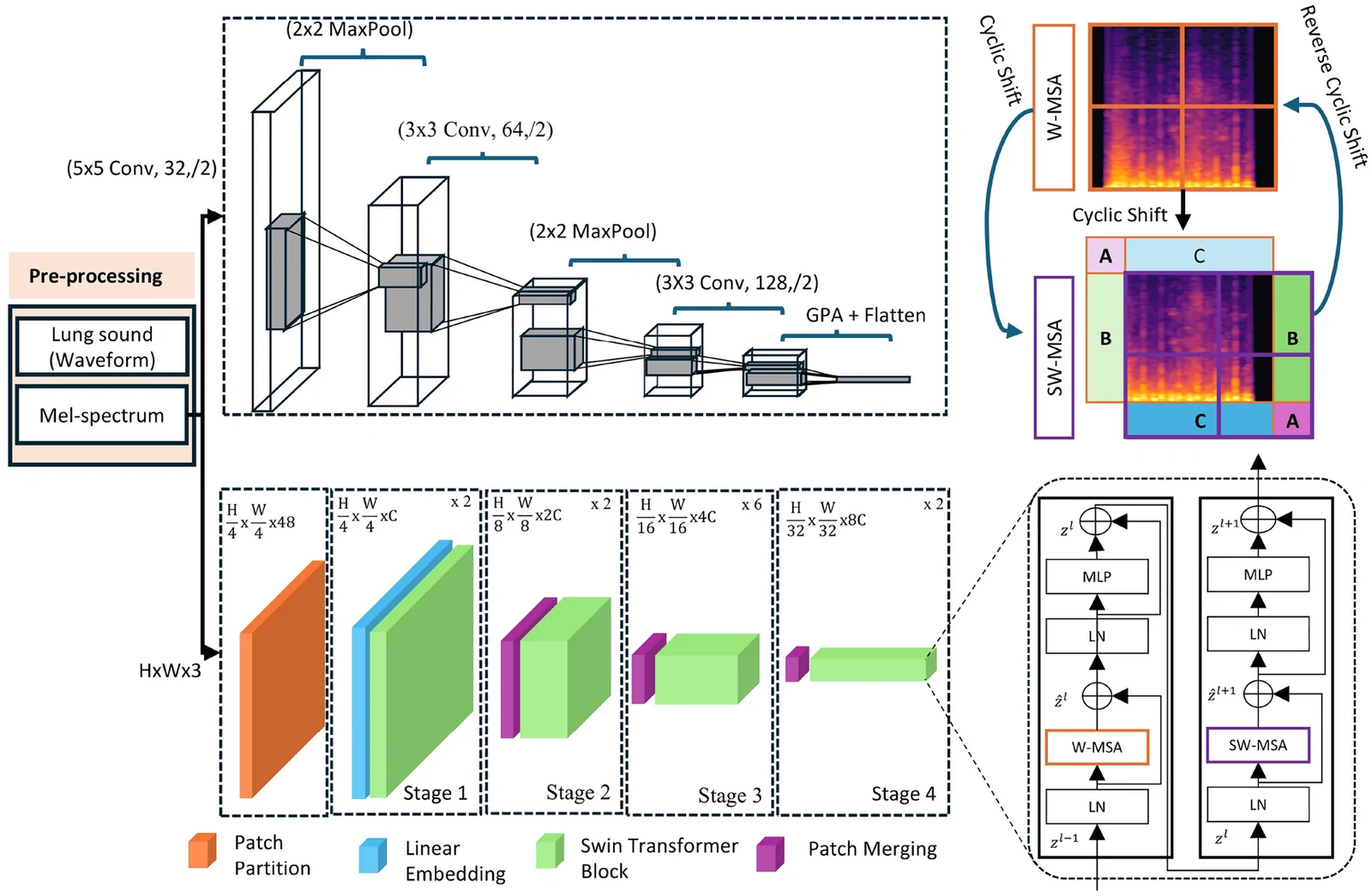
Diagram of the proposed hybrid CNN and Swin Transformer framework

### Swin transformer

The Swin Transformer is a vision transformer variant designed to extract visual features using hierarchical representation and window-based self-attention. Unlike ViT, which uses global self-attention (with quadratic computational cost) [9], Swin Transformer computes self-attention within non-overlapping local windows and enables cross-window information exchange through the shifted-window mechanism. This makes the architecture computationally efficient in image-based classification, such as respiratory sound classification [25]. In this study, the Swin-Tiny configuration is selected because it balances computational efficiency and extraction capability for the limited-size ICHBI medical datasets. The typical Swin-T uses an embedding dimension of 96 and a layer depth of (2, 2, 6, 2) across the stages. The model processes a 3-channel input spectrogram image with a window size of 7. As illustrated in Fig. 2, the Swin Transformer branch follows a four-stage hierarchical architecture with patch partitioning, linear embedding, Swin Transformer blocks, and patch-merging operations. To reduce overfitting, dropout regularization with a rate of 0.3 is used.

The Swin-T branch receives a three-channel log-mel spectrogram image $I \in \mathbb {R}^{H \times W \times 3}$ as input. The input image is then divided into non-overlapping *4 × 4* patches. Since the input has three channels, each patch has a dimension of $4 \times 4 \times 3 = 48$, resulting in an initial patch representation of $\frac{H}{4} \times \frac{W}{4} \times 48$. As shown in the figure, the linear embedding layer projects the non-overlapping patches to C channels. In each block, window-based (W-MSA) and shifted window-based multi-head self-attention (SW-MSA) are implemented. Between consecutive stages, patch-merging layers reduce spatial resolution while increasing the channel dimension from 32 *C* to 2*C*, 4*C*, and 8*C*. In standard W-MSA, after the spectrogram is divided into non-overlapping windows, self-attention is independently computed within each window. The consecutive Swin Transformer blocks are formulated as follows [25, 26]:

12 $$\begin{aligned} \hat{z}^{l}&= W\text {-}MSA\left( LN(z^{l-1})\right) + z^{l-1}, \end{aligned}$$

13 $$\begin{aligned} z^{l}&= MLP\left( LN(\hat{z}^{l})\right) + \hat{z}^{l}, \end{aligned}$$

14 $$\begin{aligned} \hat{z}^{l+1}&= SW\text {-}MSA\left( LN(z^{l})\right) + z^{l}, \end{aligned}$$

15 $$\begin{aligned} z^{l+1}&= MLP\left( LN(\hat{z}^{l+1})\right) + \hat{z}^{l+1}. \end{aligned}$$

In Equations (12)–(15), $\hat{z}^{l}$ and $\hat{z}^{l+1}$ denote the intermediate output after the W-MSA and SW-MSA modules, respectively, while $z^{l}$ and $z^{l+1}$ denote the output after the MLP modules. Here, *LN* represent layer normalization. As a result, the final feature map can be expressed as $I \in \mathbb {R}^{\frac{H}{32} \times \frac{W}{32} \times 8C}$. Through average pooling, the 8*C*dimensional representation is flattened to obtain $z_1^{(l+1)}$.

### Model architecture

Figure 1c shows the model architecture applied to the dataset after preprocessing. Although all models received the same log-Mel spectrogram input, each architecture reads it differently. 1D CNN extracts temporal feature patterns by treating the spectrogram as a multi-channel time sequence. The 2D CNN captures the local intensity variation in the time-frequency plane. The Swin Transformer is designed to model long-range dependencies through its hierarchical window-based attention mechanism. Finally, the hybrid model integrates local and global features, enabling a richer representation of respiratory sound characteristics.

### Fusion architecture

Figure 2 illustrates the detailed model structure used in the fusion architecture. For consistency, similar structures are applied to each of the single baseline models. The CNN branch also implements compact hierarchical feature extraction. Like the Swin Transformer branch, the log-Mel spectrogram image $I \in \mathbb {R}^{3 \times H \times W}$ is used in the CNN branch input and passes through consecutive convolutional blocks with *5 × 5* and *3 × 3* kernels, using the rectified linear unit (ReLU) activation function. Each convolution block is followed by a *2 × 2* max-pooling layer, which increases the semantic strength of the features while reducing the spectro-spatial resolution, thereby improving the feature extraction capability. Similar to the Swin Transformer branch, after the convolutional operations, a global average pooling layer aggregates the feature maps, followed by a fully connected head with dropout to produce a 512-dimensional representation $z_2^{(l+1)}$. Finally, the prediction is obtained by concatenating the extracted features from the Swin Transformer branch ($z_1^{(l+1)}$) and the CNN branch ($z_2^{(l+1)}$). The prediction is then evaluated by passing the concatenated dense features through a fully connected neural network.

16 $$\begin{aligned} z^{(l+1)}&= \text {concat}\left( z_1^{l+1}, z_2^{l+1}\right) \end{aligned}$$

17 $$\begin{aligned} f^{(l+1)}&= MLP\left( z^{(l+1)}\right) \end{aligned}$$

After passing through the MLP, the final classification distribution is determined by passing the resulting fused vector through the SoftMax classification layer. For Task 1, each segment is classified using the probabilistic output of the independent SoftMax classification layer. In contrast, for Task 2, the SoftMax probabilities from all segments belonging to the same recording are averaged to obtain the recording-level prediction. Furthermore, both the baseline models and the hybrid model are trained using the Adam optimizer with a batch size of 32. The models were trained using the Adam optimizer with a batch size of 32. Learning rates of $10^{-3}$ and $10^{-4}$ were used for the CNN branch and Swin Transformer branch, respectively, to ensure stable convergence. To reduce the risk of overfitting, early stopping was applied based on the validation loss. To ensure reproducibility and assess the robustness of the reported results, all experiments were repeated using five different random seeds: 0, 7, 21, 42, and 123.

## Results

### Comparison of baselines and hybrid model

**Fig. 3:**
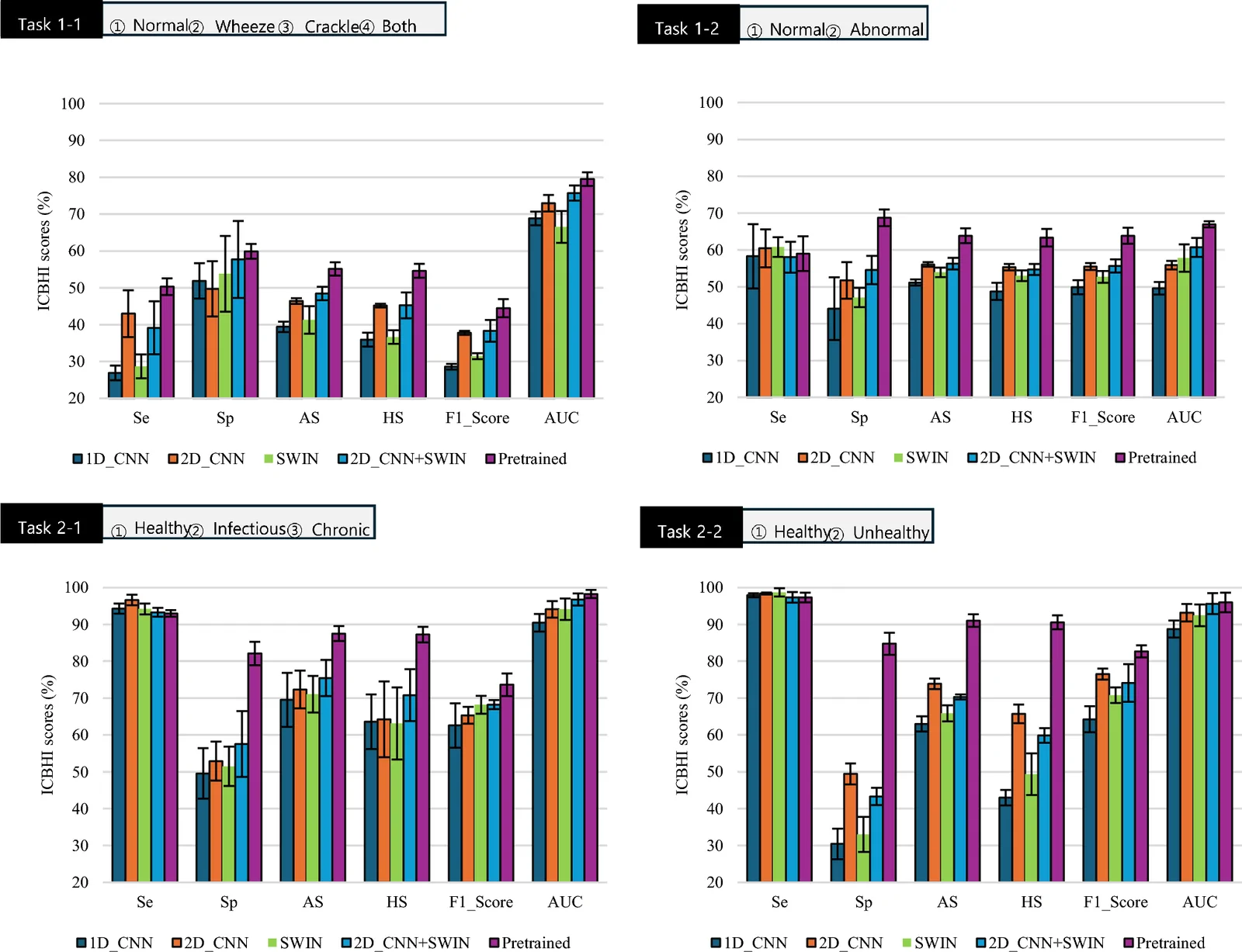
Performance comparison of 1D-CNN, 2D-CNN, SWIN Transformers, and hybrid CNN + SWIN models across the four ICBHI subtasks (**a** Task 1-1, **b** Task 1-2, **c** Task 2-1, and **d** Task 2-2). Evaluation metrics include sensitivity (Se), specificity (Sp), average score (AS), harmonic score (HS), F1-Score, and AUC curve

Task-wise performance comparisons of CNNs, transformers, and the proposed hybrid model are illustrated in Fig. 3. The results of the fusion models are compared with the single-stream baselines (1D CNN, 2D CNN, and Swin Transformer). As shown in Fig. 3a for Task 1-1, the pretrained fusion model achieved notable performance improvements over the individual baseline models. Both the 1D-CNN and Swin Transformer baseline models show limited performance across all evaluation metrics. This is due to the limited temporal feature extraction of 1D CNN and the data-intensive nature of Swin Transformers. In contrast, the 2D CNN outperforms the other single-stream baseline models as it captures spectro-spatial patterns. It even achieves competitive performance in AS, reaching $45.4 \pm 0.75\%$, compared to the non-pretrained fusion variant, which scores $48.4\pm 1.84\%$. However, when examining sensitivity, the 2D CNN shows noticeable bias and uncertainty in the anomaly-breath detection rate ($42.9\pm 6.3\%$) with higher specificity. The fusion architecture, in both its non-pretrained and pretrained variants, consistently outperforms the baseline models in most results, demonstrating a more balanced ability to detect both normal and abnormal respiratory sounds. Figure 3b presents the results for Tasks 1-2. Like the four-class case, the fusion models show improved performance compared to the individual baseline models. In all metric cases, performance is increased.

Unlike Task 1-1, both the 2D-CNN and Swin Transformer baselines performed similarly in reducing detection uncertainty. This is due to the increase in data size after class rearrangement. In this specific task, across all architectures, sensitivity and specificity remain well-proportioned due to balanced class distribution. Similar to Task 1-1, the pretrained models outperformed the non-pretrained variants in all cases, achieving the most balanced and consistently strong results across all metrics.

**Fig. 4:**
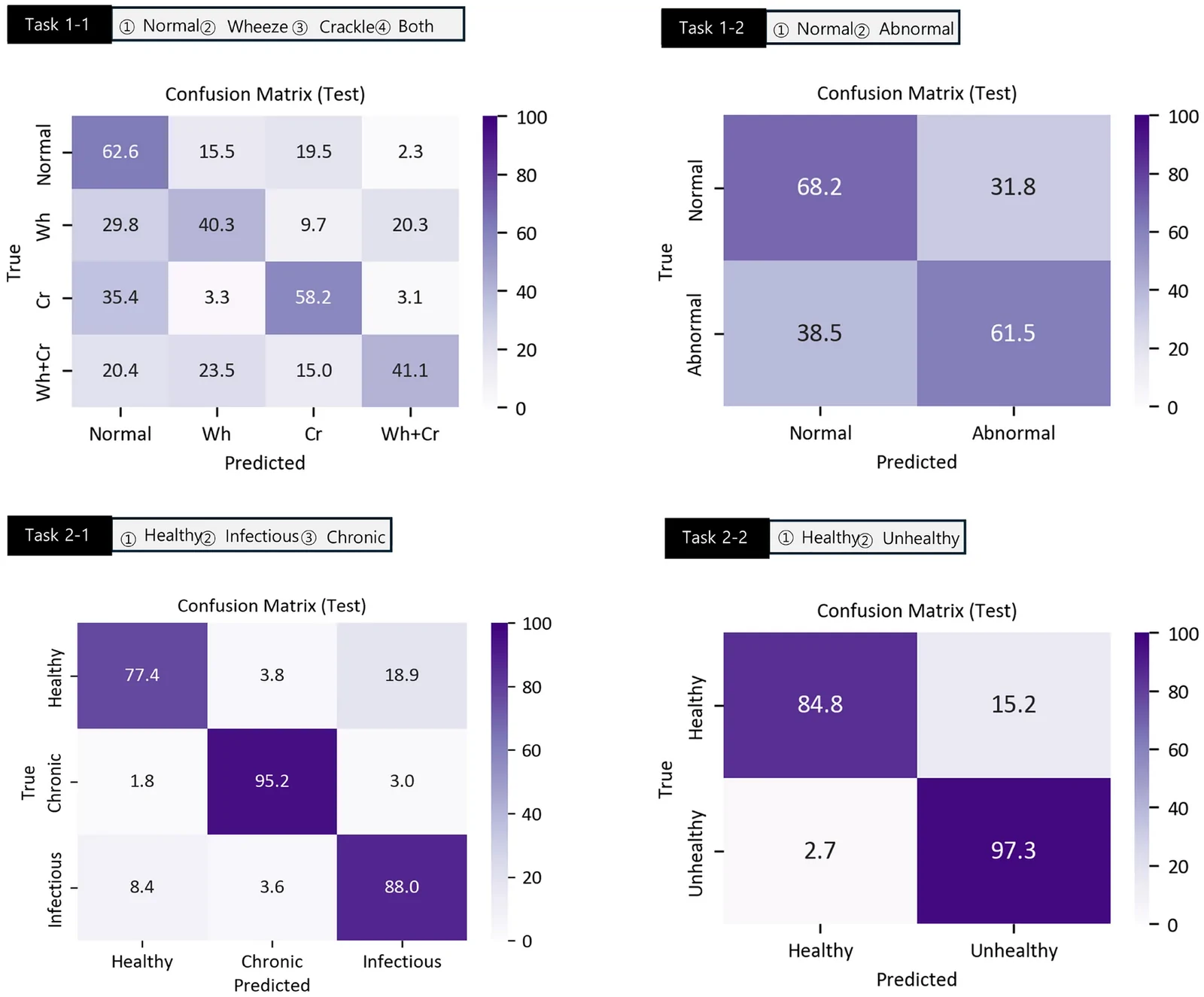
Normalized confusion matrices (%) of the proposed pretrained model across the four ICBHI subtasks: **a** Task 1-1, **b** Task 1-2, **c** Task 2-1, and **d** Task 2-2. The matrices show the average results obtained over five random seeds

The disease-classification task provides a clear setting for evaluating models under severe data imbalance, as the training set consists of 3.4%, 85.4%, and 11.2% healthy, infectious, and chronic disease recordings, respectively. In both disease-level subtasks, Task 2-1 (Fig. 3c) and Task 2-2 (Fig. 3d), sensitivity remains consistently higher than specificity. This trend is mainly due to the high proportion of unhealthy patients. Only the pretrained fusion model achieves relatively higher specificity, indicating more balanced detection compared with the baseline models. Moreover, as shown in Table 2, this performance surpasses the current state-of-the-art (SOTA) models. The pretrained fusion model exhibits a strong balance between sensitivity and specificity. As shown in the figures and depicted in Supplementary Table 1, for Task 2-1, AS and HS of $87.5\pm 2.0\%$ and $87.2\pm 2.1\%$ are observed, respectively; likewise, for Task 2-2, AS and HS of $91.0\pm 1.7\%$ and $90.6\pm 1.90\%$ are observed for the pretrained fusion model. Across all tasks and evaluation metrics, including F1-score and AUC, the pretrained hybrid model outperforms the baseline models. Moreover, across all classification tasks and evaluation metrics, including F1-score and AUC, the pretrained hybrid model consistently achieves superior performance compared with the baseline models. This finding is further supported by the ROC analysis shown in Supplementary Fig. 1, where the pretrained hybrid model generally produces ROC curves closer to the upper-left corner across the four tasks, indicating stronger discriminative capability and improved class-separation performance. These results provide additional confirmation that the pretrained CNN–Swin Transformer fusion model offers more robust and balanced diagnostic performance than the individual 1D-CNN, 2D-CNN, Swin Transformer, and non-pretrained fusion models.

**Table 2:** Comparison of the Proposed Method with State-of-the-Art Approaches on the ICBHI Dataset (60/40 Train–Test Split).

Task	Model	Year	Sp(%)	Se(%)	AS(%)	HS(%)	DBI (a.u.)
Task 1-1							
	CNN [27]	2021	73.0	32.0	53.0	45.0	0.609
	ViT [9]	2022	78.31	36.41	57.36	49.7	0.635
	Co-Tuning [3]	2022	79.3	37.2	58.3	50.6	0.638
	ResNet-34 [19]	2023	75.3	25.1	50.2	37.6	0.500
	AST [8]	2023	53.5	42.9	52.5	50.8	0.890
	AST [21]	2023	81.7	43.1	62.4	56.4	0.691
	RepAugment [28]	2024	**82.5**	40.5	61.5	54.4	0.658
	CycleGuardian [2]	2025	82.1	44.5	**63.3**	**57.7**	0.703
	CNN+SWIN [ours]	2025	81.1	43.3	48.2	43.1	0.691
	Pre-trained [ours]	2025	59.90	**50.30**	55.1	54.6	**0.913**
Task 1-2							
	Co-Tuning [3]	2022	79.3	50.1	64.7	61.3	0.774
	AST [21]	2023	**81.7**	55.8	**68.7**	66.3	0.812
	CycleGuardian [2]	2025	71.9	**62.8**	67.3	**67.1**	**0.933**
	CNN+SWIN [ours]	2025	64.3	49.4	56.9	55.9	0.869
	Pre-trained [ours]	2025	68.7	59.0	63.9	63.3	0.925
Task 2-1							
	CNN [27]	2021	**88.0**	85.0	86.0	86.0	**0.983**
	CNN-MoE [4]	2021	71.0	**98.0**	84.0	84.0	0.840
	Co-Tuning [3]	2022	81.1	90.2	85.7	85.2	0.947
	CNN+SWIN [ours]	2025	47.1	92.6	69.9	62.3	0.674
	Pre-trained [ours]	2025	82.1	93.0	**87.5**	**87.3**	0.937
Task 2-2							
	CNN-MoE [4]	2021	**98.0**	71.0	84.0	84.0	0.840
	Co-Tuning [3]	2022	84.5	95.1	**89.9**	87.1	**0.941**
	CNN+SWIN [ours]	2025	96.1	47.1	71.6	63.2	0.658
	Pre-trained [ours]	2025	84.5	**97.3**	**91.0**	**90.6**	0.930

### Pre-trained model evaluation

The proposed fusion architecture combines two pretrained backbones. The Swin Transformer branch uses Swin Transformer V2, accessed through the keras_cv_attention_models.swin_transformer_v2 library [25], which is initialized with pretrained weights on the ImageNet-1k dataset. Likewise, the CNN branch uses EfficientNet-B0 from the TensorFlow application set [29] that was pretrained on the ImageNet-1k dataset. These pretrained models give a strong initialization that enhances the classification performance. Task 1-1 (Fig. 4a) illustrates the normalized confusion matrix (in%) for the proposed pretrained fusion model; it shows that the normal respiratory cycle is more accurately identified than the other classes. In addition, the limited number of samples available for the wheeze and both (wheeze + crackle) classes results in a low true-positive rate. However, compared with the current state-of-the-art model [2], improvements of 28.55% and 14.2% are observed in true-positive detection for crackle and wheeze, respectively. However, compared with the current state-of-the-art model [2], our model shows improvements of 28.55% and 14.2% in true-positive detection for crackle and wheeze, respectively. However, our model still struggles to identify true positives for both wheeze and crackle sounds, which are frequently misclassified as either ”crackle” or ”wheeze.” Figure (b) shows the result of Task 1-2, where nearly two-thirds of the respiratory cycles are correctly classified and show a balanced metric measure in both sensitivity and specificity. Even though a notable portion of the classes are misclassified, the results indicate our model does not exhibit a bias toward either class. For Task 2-1 (Fig. 4c), the pretrained model accurately identifies ”healthy,” ”chronic,” and ”infectious” patient cases. Moreover, under the ICBHI metric evaluation–which prioritizes distinguishing healthy from unhealthy patients–our model achieves higher AS and HS than the state-of-the-art methods. In Task 2-2 (Fig. 4d), the confusion between chronic and infectious cases is effectively reduced because both categories are merged into a single unhealthy class. As a result, the model shows more stable detection performance for both healthy and unhealthy cases. Furthermore, Supplementary Table 2 provides the detailed confusion matrices of the pretrained fusion model across all classification tasks, reported as mean ± standard deviation over five random seeds.

### Comparison with existing methods

From Table 2, both our non-pretrained and pretrained fusion variants demonstrate balanced detection across sensitivity and specificity. For Task 1-1, although AS is slightly lower than SOTA, our model achieves the highest Se, addressing the key diagnostic imbalance problem. The model achieves the highest sensitivity, outperforming existing methods by 5.8%. Although the AS and HS remain below the top-performing four-class methods, the pretrained fusion still performs balanced detection. Similarly, in anomaly-level classification of Task 2-1, the pretrained fusion surpasses prior models in AS and HS ICBHI by improving 1.5% and 1.3%, respectively, and achieves competitive performance in both Sp and Se. These gains stem from the complementary strengths of hybrid architecture. Similarly, in anomaly-level classification of Task 2-2, the pretrained fusion surpasses prior models in most ICBHI metrics except Sp, improving Se, AS, and HS by 2.2%, 1.9%, and 3.5%, respectively. Overall, the pretrained CNN+Swin fusion model sets a new benchmark for balancing sensitivity and specificity while achieving results comparable to state-of-the-art models.

## Conclusion

This study addressed the diagnostic imbalance in ICBHI respiratory sound classification, particularly the unstable trade-off between sensitivity (Se) and specificity (Sp). In clinical biomedical classification, improving a single overall performance score is insufficient because a model may correctly classify normal sounds while still missing pathological respiratory events. Therefore, maintaining a balanced Se–Sp relationship is essential to reduce false or non-actionable detections while preserving clinically meaningful identification of abnormal lung sounds.

To investigate this problem, this study evaluated multiple neural network models, including a 1D-CNN, 2D-CNN, self-attention-based Swin Transformer, and both untrained and pretrained CNN–Swin Transformer fusion models, while strictly following the official ICBHI evaluation protocol. The results show that the pretrained hybrid fusion model provides more balanced diagnostic behavior by improving sensitivity while maintaining competitive specificity across multiple tasks. This advantage is mainly attributed to the model’s ability to integrate CNN-based local spectro-temporal feature extraction with Swin Transformer-based global contextual dependency modeling. Although Task 1-1 remains challenging, the proposed fusion model achieves a substantial improvement in sensitivity while maintaining competitive specificity, resulting in improved AS and HS compared with baseline models. In disease-level classification, the model also demonstrates stronger performance by better capturing pathological respiratory sound patterns. Overall, these findings indicate that the pretrained CNN–Swin Transformer fusion framework is an effective approach for stabilizing the sensitivity–specificity trade-off in noisy and imbalanced lung sound classification, providing a promising foundation for future clinical decision-support systems in automatic auscultation.

Building on this foundation, we recommend that future work move beyond architectural improvements toward clinically validated deployment. Key clinical requirements include minimizing diagnostic bias and improving robustness under real-world acoustic conditions. This can be achieved by developing architectures that balance sensitivity and specificity, evaluating performance on larger and more heterogeneous datasets, and adopting clinically aligned evaluation protocols. Furthermore, while the proposed model leverages a combination of CNN- and Transformer-based models, the two architectures are fused at a late feature level using concatenation. This limits deep interaction between the CNN and Swin Transformer at early or intermediate stages, which restricts the full potential of multimodal fusion. Future work may therefore investigate early- or intermediate-level fusion strategies, as well as more advanced mechanisms such as gated fusion, attention-based fusion, cross-attention, and feature alignment to enable deeper and more effective feature interaction.

## Supplementary Information

Below is the link to the electronic supplementary material.

Supplementary Material 1.

## Data Availability

Data will be made available on request.
