# Supplementary material for: Fusion of CNN and swin transformer for robust lung sound classification
Source: Biomed Eng Lett. 2026 Sep 23;16(6):1579–91. doi: 10.1007/s13534-026-00616-6 (PMC13740432; doi:10.1007/s13534-026-00616-6)
Supplement: Supplementary file 1 [file 13534_2026_616_MOESM1_ESM.docx]

**Supplementary Fig.1**

①

②

④

③

Both

Task 1-1

Normal

Wheeze

Crackle

①

②

Task 1-2

Normal

Abnormal

①

②

③

Task 2-1

Healthy

Infectious

Chronic

①

②

Task 2-2

Healthy

Unhealthy

**Supplementary Fig. 1:** Representative ROC curve comparison of 1D-CNN, 2D-CNN, Swin Transformer, 2D-CNN+Swin fusion, and pretrained fusion models across Tasks 1-1, 1-2, 2-1, and 2-2 using seed 42.

**Supplementary Table 1:** Classification performance of all models across Tasks 1-1, 1-2, 2-1, and 2-2 reported as mean ± std.

|  | **Model** | **Se** | **Sp** | **AS** | **HS** | **F1_Score** | **AUC** |
| --- | --- | --- | --- | --- | --- | --- | --- |
| **Task 1-1** | 1D_CNN | 26.90 ± 1.98 | 51.88 ± 4.78 | 39.40 ± 1.42 | 35.92 ± 1.88 | 28.58 ± 0.77 | 68.84 ± 1.85 |
|  | 2D_CNN | 42.98 ± 6.34 | 49.72 ± 7.50 | 46.40 ± 0.75 | 45.18 ± 0.48 | 37.78 ± 0.55 | 72.95 ± 2.24 |
|  | SWIN | 28.69 ± 3.24 | 53.80 ± 10.29 | 41.26 ± 3.74 | 36.64 ± 1.86 | 31.46 ± 0.81 | 66.52 ± 4.33 |
|  | 2D_CNN+SWIN | 39.14 ± 7.19 | 57.70 ± 10.43 | 48.46 ± 1.85 | 45.24 ± 3.53 | 38.32 ± 2.93 | 75.73 ± 2.04 |
|  | Pretrained | 50.30 ± 2.26 | 59.90 ± 2.03 | 55.12 ± 1.81 | 54.62 ± 1.88 | 44.46 ± 2.45 | 79.50 ± 1.84 |
| **Task 1-2** | 1D_CNN | 58.26 ± 8.73 | 44.08 ± 8.52 | 51.16 ± 0.83 | 48.76 ± 2.32 | 49.88 ± 1.91 | 49.59 ± 1.69 |
|  | 2D_CNN | 60.44 ± 5.16 | 51.74 ± 4.96 | 56.08 ± 0.64 | 55.32 ± 0.89 | 55.52 ± 0.93 | 55.87 ± 1.16 |
|  | SWIN | 60.80 ± 2.68 | 47.10 ± 2.62 | 53.92 ± 1.29 | 52.98 ± 1.45 | 52.68 ± 1.60 | 57.80 ± 3.72 |
|  | 2D_CNN+SWIN | 58.04 ± 4.20 | 54.56 ± 3.84 | 56.28 ± 1.59 | 54.68 ± 1.49 | 55.66 ± 1.80 | 60.68 ± 2.58 |
|  | Pretrained | 59.00 ± 4.71 | 68.72 ± 2.28 | 63.86 ± 2.00 | 63.32 ± 2.38 | 63.86 ± 2.17 | 66.94 ± 0.83 |
| **Task 2-1** | 1D_CNN | 94.30 ± 1.37 | 49.56 ± 6.85 | 69.52 ± 7.33 | 63.58 ± 7.40 | 62.58 ± 6.03 | 90.44 ± 2.39 |
|  | 2D_CNN | 96.65 ± 1.42 | 52.91 ± 5.29 | 72.36 ± 5.12 | 64.25 ± 10.26 | 65.36 ± 2.29 | 94.09 ± 2.26 |
|  | SWIN | 94.20 ± 1.48 | 51.50 ± 5.35 | 71.06 ± 4.97 | 63.14 ± 9.78 | 68.18 ± 2.43 | 94.14 ± 2.92 |
|  | 2D_CNN+SWIN | 93.30 ± 1.20 | 57.54 ± 8.93 | 75.45 ± 4.92 | 70.81 ± 7.05 | 68.24 ± 1.23 | 96.78 ± 1.65 |
|  | Pretrained | 92.98 ± 0.89 | 82.11 ± 3.19 | 87.53 ± 2.04 | 87.25 ± 2.12 | 73.62 ± 3.08 | 98.26 ± 1.10 |
| **Task 2-2** | 1D_CNN | 97.92 ± 0.53 | 30.42 ± 4.14 | 62.99 ± 2.06 | 42.98 ± 2.11 | 64.26 ± 3.54 | 88.75 ± 2.30 |
|  | 2D_CNN | 98.40 ± 0.27 | 49.41 ± 2.85 | 73.90 ± 1.44 | 65.73 ± 2.55 | 76.52 ± 1.49 | 93.18 ± 2.38 |
|  | SWIN | 98.70 ± 1.13 | 33.00 ± 4.75 | 65.88 ± 2.18 | 49.36 ± 5.64 | 70.80 ± 2.11 | 92.46 ± 2.95 |
|  | 2D_CNN+SWIN | 97.34 ± 1.43 | 43.32 ± 2.35 | 70.30 ± 0.68 | 59.86 ± 1.98 | 74.12 ± 5.11 | 95.64 ± 2.85 |
|  | Pretrained | 97.31 ± 1.30 | 84.75 ± 2.98 | 91.04 ± 1.70 | 90.58 ± 1.89 | 82.68 ± 1.64 | 95.96 ± 2.64 |

**Supplementary Table 2:** Normalized prediction performance (%) of the proposed pretrained fusion model across the four ICBHI subtasks, reported as mean ± std across multi-seed experiments.

|  | Actual /  Predicted | Normal | Wheeze | Crackle | Wheeze + Crackle |
| --- | --- | --- | --- | --- | --- |
| **Task 1-1** | Normal | 62.60 ± 2.39 | 15.54 ± 0.58 | 19.52 ± 8.18 | 2.33 ± 0.21 |
|  | Wheeze | 29.82 ± 2.17 | 40.26 ± 2.69 | 9.66 ± 0.45 | 20.26 ± 0.75 |
|  | Crackle | 35.44 ± 0.97 | 3.27 ± 0.37 | 58.24 ± 1.98 | 3.05 ± 0.66 |
|  | Wheeze + Crackle | 20.42 ± 2.27 | 23.50 ± 0.71 | 14.97 ± 1.13 | 41.12 ± 2.63 |

| **Task 2-1** | Actual /  Predicted | Healthy | Chronic | Infectious |
| --- | --- | --- | --- | --- |
|  | Healthy | 77.36 ± 7.88 | 3.77 ± 3.54 | 18.87 ± 12.31 |
|  | Chronic | 1.78 ± 1.09 | 95.22 ± 1.60 | 3.01 ± 2.26 |
|  | Infectious | 8.43 ± 15.42 | 3.61 ± 4.82 | 87.95 ± 26.14 |

| **Task 1-2** | Actual /  Predicted | Normal | Abnormal |
| --- | --- | --- | --- |
|  | Normal | 68.23 ± 1.8 | 31.77 ± 1.92 |
|  | Abnormal | 38.53 ± 4.18 | 61.47 ± 3.58 |

| **Task 2-2** | Actual /  Predicted | Healthy | Unhealthy |
| --- | --- | --- | --- |
|  | Healthy | 84.76 ± 9.5 | 15.24 ± 10.12 |
|  | Unhealthy | 2.69 ± 1.24 | 97.31 ± 1.5 |
